# Development and validation of nomogram prediction model for diabetic hearing impairment based on the levels of lncRNA MALAT1, miR-199b and AGEs in peripheral blood

**DOI:** 10.3389/fendo.2025.1573661

**Published:** 2025-09-10

**Authors:** Di Bian, Haoran Li, Le Wang, Yujuan Chen, Zhaobing Qin

**Affiliations:** ^1^ Department of Otology, The First Affiliated Hospital of Zhengzhou University, Zhengzhou, China; ^2^ Department of Otolaryngology, The Fifth Affiliated Hospital of Zhengzhou University, Zhengzhou, China; ^3^ Department of Geriatric Endocrinology, The First Affiliated Hospital of Zhengzhou University, Zhengzhou, China

**Keywords:** diabetic hearing impairment, lncRNA MALAT1, miR-199b, advanced glycation end products, nomogram

## Abstract

**Objective:**

To develop a nomogram prediction model for diabetic hearing impairment (DHI) based on peripheral blood long noncoding RNA *MALAT1*(lncRNA MALAT1), microRNA-199b (miR-199b) and advanced glycation end products (AGEs), and to evaluate its clinical utility. These biomarkers were selected due to their established roles in diabetes-related complications, including oxidative stress, inflammation, and vascular dysfunction, which are implicated in hearing impairment.

**Methods:**

This cross-sectional study included 318 diabetic patients without pre-existing hearing loss, who were randomly divided into a training set (n=223) and a validation set (n=95) at a ratio of 7:3. Peripheral blood levels of LncRNA MALAT1 and miR-199b were quantified using real-time fluorescence quantitative PCR (RT-qPCR), and AGEs were measured by enzyme-linked immunosorbent assay (ELISA). Pure-tone audiometry (PTA) was performed to diagnose hearing impairment. Multivariate logistic regression identified independent influencing factors, and a prediction model in the form of nomogram is constructed accordingly. In order to evaluate the prediction accuracy of the model, the operating characteristic curve (ROC curve) and calibration curve of the subjects were further drawn. Decision curve analysis (DCA) was adopted to comprehensively evaluate the significance and value of the nomogram model.

**Results:**

There was no significant difference in baseline characteristics between training and validation sets (all *P*>0.05). In the training set, diabetic peripheral neuropathy, high levels of AGEs, fasting blood glucose (FBG), postprandial blood glucose (2hPG), high expression of lncRNA *MALAT1*, low expression of miR-199b and long course of diabetes were independent risk factors for diabetic hearing impairment (all *P*<0.05). The nomogram exhibited good discriminative ability, with areas under the curve (AUC) of ROC curve of 0.810 (95% *CI*: 0.737-0.883) (training set) and 0.739 (95% *CI*: 0.597-0.882) (validation set), confirming predictive accuracy of the model. Calibration was confirmed by Hosmer-Lemeshow tests (*P*=0.610 and *P*=0.534, respectively), indicating no significant deviation from perfect fit.

**Conclusion:**

The nomogram integrates clinical and biomarker data to predict DHI risk, offering a practical tool for early intervention.

## 1 Introduction

Diabetes is a chronic metabolic disease characterized by abnormally elevated blood glucose levels, mainly driven by insufficient insulin secretion and/or impaired insulin action, leading to disorders of carbohydrate, fat and protein metabolism. According to the International Diabetes Federation (IDF), the global number of diabetic patients is rising continuously, with type 2 diabetes accounting for over 90% by 2045, and this study focuses on type 2 diabetic patients (1). A common complication, diabetic hearing impairment (DHI), seriously affects patients’ quality of life, manifesting as hearing loss, tinnitus, auditory hyperesthesia, and progressive aggravation (2–4). Its pathogenesis involves metabolic dysfunction, oxidative stress, inflammatory activation, and vascular pathology, particularly damaging key auditory structures like the cochlear stria vascularis and spiral ganglion cells.

Long noncoding RNAs (lncRNAs) regulate gene transcription, cell differentiation, proliferation, and apoptosis (5). lncRNA *MALAT1*, highly expressed in tumors and diabetic complications, regulates cell proliferation, migration and invasion (6). Studies have shown that expression of MALAT1 was significantly upregulated in the cochlear tissues of diabetic patients (7). It can promote the release of IL-6 and TNF-α from spiral ganglion cells by activating the *NF-κB* inflammatory pathway, leading to demyelination of nerve fibers, which is directly related to the pathological feature of slowed auditory nerve conduction velocity in diabetic hearing impairment (8). MicroRNAs (miRNAs) regulate gene expression post-transcriptionally. mir-199b, highly expressed in cochlear tissues, modulates cell proliferation, apoptosis, and differentiation; its downregulation in diabetes weakens inhibition of pro-inflammatory factors (*TNF-α*, *IL-6*) (9), contributing to DHI. Advanced glycation end products (AGEs), generated through non-enzymatic glycation, accumulate in cochlear tissues where they activate oxidative stress and inflammatory pathways via receptor for advanced glycation end products (RAGE) signaling (7). This process impairs stria vascularis function, representing a critical mechanism in DHI pathogenesis (10).

AGEs induce vascular damage, *MALAT1* promotes neural apoptosis, and miR-199b regulates inflammation—with no prior study combining them in prediction models. A nomogram, a visual tool integrating risk factors via scoring, enables intuitive risk calculation. At present, studies have developed a prediction model of diabetic hearing impairment based on clinical indicators (such as blood sugar and course of disease) with low AUC (11). In this study, the peripheral blood biomarkers (LncRNA MALAT1, miR-199b, AGEs) and clinical factors were integrated for the first time, aiming at improving the prediction efficiency and filling the gap in the application of biomarkers in this field.

## 2 Materials and methods

### 2.1 Study population

318 diabetic patients who received medical services in our hospital from January 2021 to February 2024, were selected as the subjects of this study. Inclusion criteria: (1) Meet the diagnostic criteria of diabetes established by the World Health Organization (WHO) (6). (2) The age is 40 ~ 80 years old. (3) No obvious history of hearing loss. (4) There are no other diseases that may affect hearing (such as otitis media and sudden deafness). Exclusion criteria: (1) Previous history of ear diseases (such as otitis media and otosclerosis), head injury or noise exposure. (2) Complicated with serious cardiovascular and cerebrovascular diseases, liver and renal insufficiency, malignant tumor. (3) Used drugs that may affect hearing in the past 3 months. (4) Unable to cooperate to complete the hearing test or blood sample collection. These patients were randomly divided into a training set (n=223) and a validation set (n=95) at a ratio of 7:3 using the random number table method. All patients signed an informed consent form to voluntarily participate in this study, which was reviewed and approved by the hospital ethics Committee.

### 2.2 Data collection

Collect clinical information of patient, including general information: age, sex, height, weight, family history of diabetes, family history of hearing impairment, smoking history, drinking history, hypertension, hyperlipidemia, insulin treatment, diabetes course, diabetic peripheral neuropathy, diabetic retinopathy, systolic blood pressure (SBP), diastolic blood pressure (DBP). Laboratory indexes: fasting blood glucose (FBG), postprandial blood glucose (2hPG), glycosylated hemoglobin (HbA1c), total cholesterol (TC), triglyceride (TG), low-density lipoprotein cholesterol (LDL-C) and high-density lipoprotein cholesterol (HDL-C).

### 2.3 Detection of lncRNA *MALAT1*, miR-199b and AGEs

All patients took 5mL of elbow venous blood on an empty stomach in the morning, and quickly transferred the collected blood samples to a special test tube pre-added with EDTA anticoagulant, and centrifuged at a speed of 3000rpm for 10min. The separated plasma was transferred to a sterile and precooled freezing tube, and then immediately placed in an ultra-low temperature refrigerator at -80°C for low-temperature storage for subsequent detection and analysis. The expression levels of lncRNA *MALAT1* and miR-199b in peripheral blood were quantified using real-time quantitative polymerase chain reaction (RT-qPCR). Reactions were performed on an Applied Biosystems 7500 instrument (Thermo Fisher Scientific) with SYBR Green detection, using the following parameters: threshold cycle (Ct) defined as the cycle number at which the fluorescence signal exceeded 10× the baseline standard deviation. U6 small nuclear RNA served as the endogenous control, and relative gene expression was calculated via the 2^(−ΔΔCt) method. qPCR primer sequences: (1) lncRNA *MALAT1*: Upstream: 5’-GCTGGTGGTGGTGGTGTT-3’, Downstream: 5’-CAGGGTGGTGGTGGTGAT-3’. (2) miR-199b: Upstream: 5’-ACAGTAGTTCTGATTGTCG-3’, Downstream: 5’-GTGCAGGGTCCGAGGT-3’. (3) U6: Upstream: 5’-CTCGCTTCGGCAGCACA-3’, Downstream: 5’-AACGCTTCACGAATTTGCGT-3’. PCR conditions: 95 °C pre-denaturation for 10min; 95 °C denaturation for 15s, 60 °C annealing for 30s, 72 °C extension for 30s, 40 cycles in total. Relative expression was calculated using the 2^(-ΔΔCt) method with U6 as the reference gene. Serum levels of AGEs were quantified using a commercially available enzyme-linked immunosorbent assay (ELISA) kit (R&D Systems) according to the manufacturer’s protocol. Absorbance was measured using a Bio-Rad iMark microplate reader.

All qPCR and ELISA assays were performed in triplicate, and the intra-assay coefficient of variation (CV) was calculated to evaluate detection stability. Results showed that the intra-assay CV of qPCR was <5% and that of ELISA was <8%, both meeting experimental requirements.

### 2.4 Audiometric measures

All patients received behavioral pure tone audiometry, which was performed in a soundproof room using Interacoustics AC40 audiometer. The testing frequencies included 0.5kHz, 1kHz, 2kHz, and 4kHz. These frequencies are the key frequencies for evaluating speech communication, which is in line with the clinical routine detection standards. According to the WHO 2021 Hearing Classification Standard (8), hearing impairment was defined as a pure-tone average (PTA) ≥ 20dB/HL. Based on PTA results, all patients were further stratified into: (1) the hearing impairment group (DHI group, defined as average hearing threshold ≥20 dB HL), and (2) the non-hearing impairment group (non-DHI group, average hearing threshold <20 dB HL).

### 2.5 Statistical analysis

SPSS 26.0 and R 4.3.0 software were used for data processing. For categorical variables, data are presented as frequencies and percentages (n, %). Group comparisons were performed using the *χ*² test or Fisher’s exact test, as appropriate based on expected cell frequencies. For normally distributed continuous variables, data are expressed as mean ± standard deviation, and between-group differences were assessed using independent samples t-tests. Non-normally distributed continuous variables were summarized as median (interquartile range) and analyzed using the Mann-Whitney U test. In the training set, potential risk factors associated with DHI were first screened via univariate analysis (*P*<0.05), then included in multivariate Logistic regression analysis to identify independent influencing factors of DHI, which were eventually included in the nomogram (**Table 1**). The subjects were randomly divided into the training set and validation set at a 7:3 ratio using the ‘caret’ package of R software. The “rms” package in R software was applied to development a nomogram model. The construction of the model referred to the method of Zhang et al. (11) in constructing the prognostic model for sudden sensorineural hearing loss. The “pROC” package was used to draw the receiver operating characteristic curve (ROC) for analyzing the predictive value of the model. The Bootstrap method was used for internal validation of the model, and the calibration curve of the predicted results and the actual results was drawn. The concordance index (C - index) of the model was calculated, and the Hosmer - Lemeshow test was used to evaluate the goodness - of - fit of the prediction model. Decision Curve Analysis (DCA) was used to evaluate the clinical application value of the model. Significance testing was performed with the use of a two‐sided alpha level of 0.05.

**Table 1:** Variable assignment method.

Variable	Meaning	Evaluation
X1	Diabetic peripheral neuropathy	None =0, Yes =1.
X2	Course of diabetes mellitus	continuous variable
X3	FBG	continuous variable
X4	2hPG	continuous variable
X5	lncRNA MALAT1	continuous variable
X6	miR-199b	continuous variable
X7	AGEs	continuous variable
Y	Do you have DHI?	None =0, Yes =1.

## 3 Results

### 3.1 Comparison of baseline characteristics between training set and validation set

The training and validation sets were divided using simple random sampling, with 223 patients assigned to the training set (for model construction) and 95 to the validation set (for model validation) at a 7:3 ratio from 318 patients. There was no significant difference in baseline characteristics between the training set and the validation group (all *P*>0.05) (**Table 2**).

**Table 2:** Comparison of clinical data between training set and validation set.

Indicators			Training set (n=223)	Validation set (n=95)	t/χ²/U	*P*
Age (years)			56.24 ± 8.54	55.64 ± 9.02	0.563	0.573
Gender		Male	120 (53.81)	50 (52.63)	0.037	0.846
		Female	103 (46.19)	45 (47.37)		
BMI (kg/m^2^)			22.16 ± 2.53	22.30 ± 2.37	0.460	0.645
Family history of diabetes		Yes	85 (38.12)	35 (36.84)	0.046	0.830
		No	138 (61.88)	60 (63.16)		
Family history of hearing impairment		Yes	30 (13.45)	12 (12.63)	0.039	0.843
		No	193 (86.55)	83 (87.37)		
Smoking history		Yes	45 (20.18)	20 (21.05)	0.031	0.859
		No	178 (79.82)	75 (78.95)		
Drinking history		Yes	38 (17.04)	20 (21.05)	0.719	0.396
		No	185 (82.96)	75 (78.95)		
Complicated with hypertension		Yes	71 (31.84)	31 (32.63)	0.019	0.889
		No	152 (68.16)	64 (67.37)		
Complicated with hyperlipidemia		Yes	62 (27.80)	25 (26.32)	0.074	0.785
		No	161 (72.20)	70 (73.68)		
Insulin therapy		Yes	90 (40.36)	40 (42.11)	0.084	0.771
		No	133 (59.64)	55 (57.89)		
Diabetic peripheral neuropathy		Yes	56 (25.11)	22 (23.16)	0.137	0.710
		No	167 (74.89)	73 (76.84)		
Diabetic retinopathy (DR)		Yes	48 (21.52)	21 (22.11)	0.013	0.908
		No	175 (78.48)	74 (77.89)		
Course of diabetes mellitus (year)			8.36 ± 3.49	8.45 ± 3.58	0.208	0.834
FBG (mmol/L)			10.35 ± 3.04	11.08 ± 3.12	1.944	0.052
2hPG (mmol/L)			12.34 ± 3.51	12.09 ± 3.73	0.570	0.568
HbA1c (%)			7.54 ± 1.25	7.42 ± 1.33	0.768	0.442
TC (mmol/L)			5.15 ± 1.08	5.07 ± 1.11	0.599	0.549
TG (mmol/L)			2.18 ± 0.81	2.07 ± 0.80	1.112	0.266
LDL-C (mmol/L)			2.15 ± 0.35	2.13 ± 0.41	0.442	0.658
HDL-C (mmol/L)			1.24 ± 0.32	1.29 ± 0.37	1.215	0.224
SBP (mmHg)			136.48 ± 15.05	134.85 ± 15.83	0.870	0.384
DBP (mmHg)			84.28 ± 10.05	83.87 ± 9.89	0.334	0.738
lncRNA MALAT1			1.35 ± 0.34	1.37 ± 0.36	0.471	0.637
miR-199b			0.87 ± 0.21	0.85 ± 0.24	0.774	0.457
AGEs (U/mL)			124.35 ± 24.79	123.87 ± 25.07	0.157	0.874

### 3.2 Analysis of risk factors of diabetic hearing impairment

Among 223 patients in the training set, 72 cases (32.29%) were diagnosed with DHI. In the validation set of 95 patients, 31 cases (32.63%) were diagnosed with DHI. Univariate analysis showed that diabetic peripheral neuropathy, course of diabetes mellitus, FBG, 2hPG, lncRNA *MALAT1*, miR-199b and AGEs were significantly different among different groups (all *P*<0.05) (**Table 3**). Taking the occurrence of DHI as the dependent variable (0= No, 1 = Yes), and taking the factors with significant differences in univariate analysis as covariables (**Table 1**), multivariate Logistic regression analysis was conducted. In the regression model, the tolerance of each variable is greater than 0.1, the variance expansion factor (VIF) is less than 10, and the conditional index is less than 30, and there is no same eigenvalue. The results showed the diabetic peripheral neuropathy, high levels of AGEs, FBG and 2hPG, high expression of lncRNA *MALAT1*, low expression of miR-199b and long course of diabetes are independent risk factors for DHI (all *P*<0.05) (**Table 4**). In multivariate Logistic regression analysis, adjustment for hyperlipidemia (LDL-C, HDL-C) and hypertension was added, and results showed that the original independent risk factors remained statistically significant (*P*<0.05).

**Table 3:** Univariate analysis of diabetic hearing impairment.

Indicators		Hearing impairment group (n=72)	Non-hearing impairment group (n=151)	*t*/*χ*²/U	*P*
Age (years)		55.34 ± 9.24	54.25 ± 8.56	0.866	0.387
Gender	Male	41 (56.94)	80 (52.98)	0.308	0.578
	Female	31 (43.06)	71 (47.02)		
BMI (kg/m^2^)		22.64 ± 3.15	22.85 ± 2.78	0.504	0.614
Family history of diabetes	Yes	30 (41.67)	55 (36.42)	0.568	0.451
	No	42 (58.33)	96 (63.57)		
Family history of hearing impairment	Yes	18 (25.00)	30 (19.87)	0.760	0.383
	No	54 (75.00)	121 (80.13)		
Smoking history	Yes	15 (20.83)	30 (19.87)	0.028	0.866
	No	57 (79.17)	121 (80.13)		
Drinking history	Yes	11 (15.28)	26 (17.22)	0.132	0.715
	No	61 (84.72)	125 (82.78)		
Complicated with hypertension	Yes	28 (38.89)	42 (27.81)	2.776	0.095
	No	44 (61.11)	109 (72.19)		
Complicated with hyperlipidemia	Yes	25 (34.72)	41 (27.15)	1.340	0.246
	No	47 (65.28)	110 (72.85)		
Insulin therapy	Yes	32 (44.44)	58 (38.41)	0.737	0.390
	No	40 (55.56)	93 (61.59)		
Diabetic peripheral neuropathy	Yes	30 (41.67)	35 (23.18)	8.069	**0.004**
	No	42 (58.33)	116 (76.82)		
Diabetic retinopathy (DR)	Yes	22 (30.56)	36 (23.84)	1.142	0.285
	No	50 (69.44)	115 (76.16)		
Course of diabetes mellitus (year)		10.54 ± 5.03	8.73 ± 4.76	2.606	**0.009**
FBG (mmol/L)		9.11 ± 2.54	8.21 ± 2.13	2.768	**0.006**
2hPG (mmol/L)		14.75 ± 3.34	13.51 ± 3.15	2.695	**0.007**
HbA1c (%)		8.31 ± 1.42	8.05 ± 1.59	1.180	0.238
TC (mmol/L)		5.84 ± 1.20	5.63 ± 1.23	1.201	0.230
TG (mmol/L)		2.62 ± 1.12	2.43 ± 1.08	1.213	0.226
LDL-C (mmol/L)		3.84 ± 1.07	3.66 ± 1.06	1.182	0.238
HDL-C (mmol/L)		1.16 ± 0.37	1.22 ± 0.35	1.175	0.241
SBP (mmHg)		142.08 ± 18.24	138.82 ± 17.46	1.285	0.200
DBP (mmHg)		85.46 ± 10.84	83.74 ± 10.29	1.147	0.252
lncRNA MALAT1		1.24 ± 0.48	1.04 ± 0.43	3.126	**0.002**
miR-199b		0.67 ± 0.18	0.76 ± 0.21	3.128	**0.002**
AGEs (U/mL)		152.04 ± 30.17	141.64 ± 28.83	2.481	**0.013**

The difference between groups was statistically significant (P < 0.05).

**Table 4:** Multivariate logistic regression analysis of diabetic hearing impairment.

Indicators	*B*	*Standard error*	*Wald*	*P*	*OR*	95% confidence interval
Diabetic peripheral neuropathy	1.213	0.358	11.483	0.001	3.364	1.668~6.786
Course of diabetes mellitus	0.086	0.033	6.796	0.009	1.090	1.022~1.163
FBG	0.212	0.072	8.582	0.003	1.236	1.073~1.424
2hPG	0.150	0.053	8.199	0.004	1.162	1.049~1.288
lncRNA MALAT1	1.151	0.384	8.994	0.003	3.162	1.490~6.709
miR-199b	-2.183	0.857	6.481	0.011	0.113	0.021~0.605
AGEs	0.014	0.006	5.604	0.018	1.014	1.002~1.026
constant	-7.683	1.669	20.456	0.001	0.001	

### 3.3 Development of nomogram prediction model

**Figure 1:**
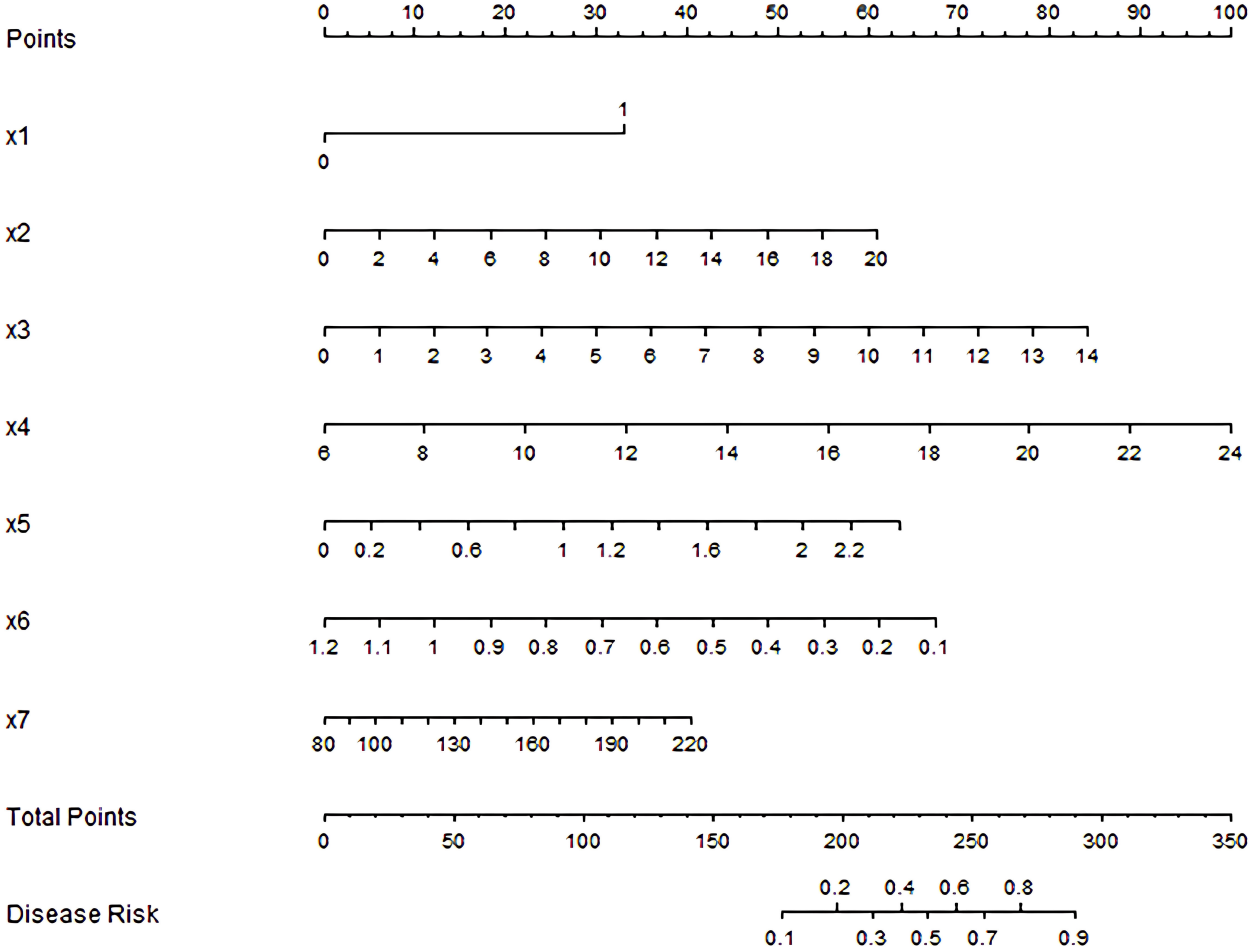
Nomogram prediction model of diabetic hearing impairment. x1: Diabetic peripheral neuropathy, x2: Course of diabetes mellitus, x3: FBG, x4: 2hPG, x5: lncRNA MALAT1, x6: miR-199b, x7: AGEs.

Based on the results of multivariate Logistic regression analysis, a nomogram prediction model for diabetes-related hearing impairment was constructed (**Figure 1**). In the nomogram, each risk factor is given a specific scale line segment. By accurately positioning the actual value of each risk factor on the corresponding scale line segment, and then projecting vertically, the corresponding score value of each risk factor can be obtained. The sum of these scores is the total score, and the predicted probability value corresponding to the total score represents the specific risk level of patients with diabetes-related hearing impairment. Example of nomogram use: A 55-year-old male with type 2 diabetes, 10-year disease duration, peripheral neuropathy, FBG 9.5 mmol/L, 2hPG 15 mmol/L, lncRNA MALAT1 1.3, miR-199b 0.6, AGEs 150 U/mL. Scores in the nomogram: peripheral neuropathy (32.5), 10-year duration (25), FBG 9.5 (57.5), 2hPG 15 (50), MALAT1 1.2 (30), miR-199b 0.6 (35), AGEs 150 (20). Total score=250, corresponding to a predicted probability of DHI of ~65%.

### 3.4 Evaluation and validation of nomogram prediction model

**Figure 2:**
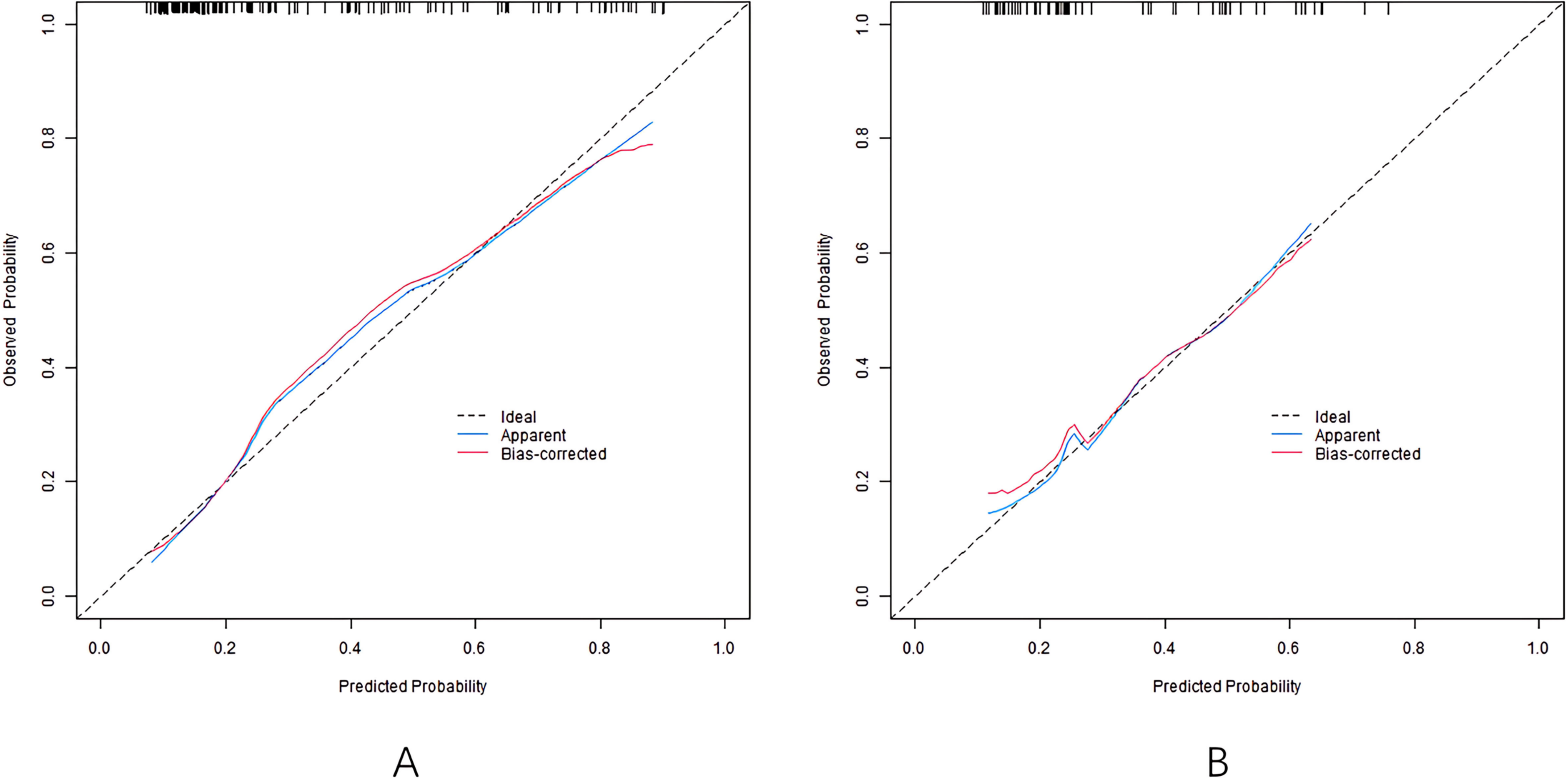
Calibration curves in the training set **(A)** and the validation set **(B)**.

**Figure 3:**
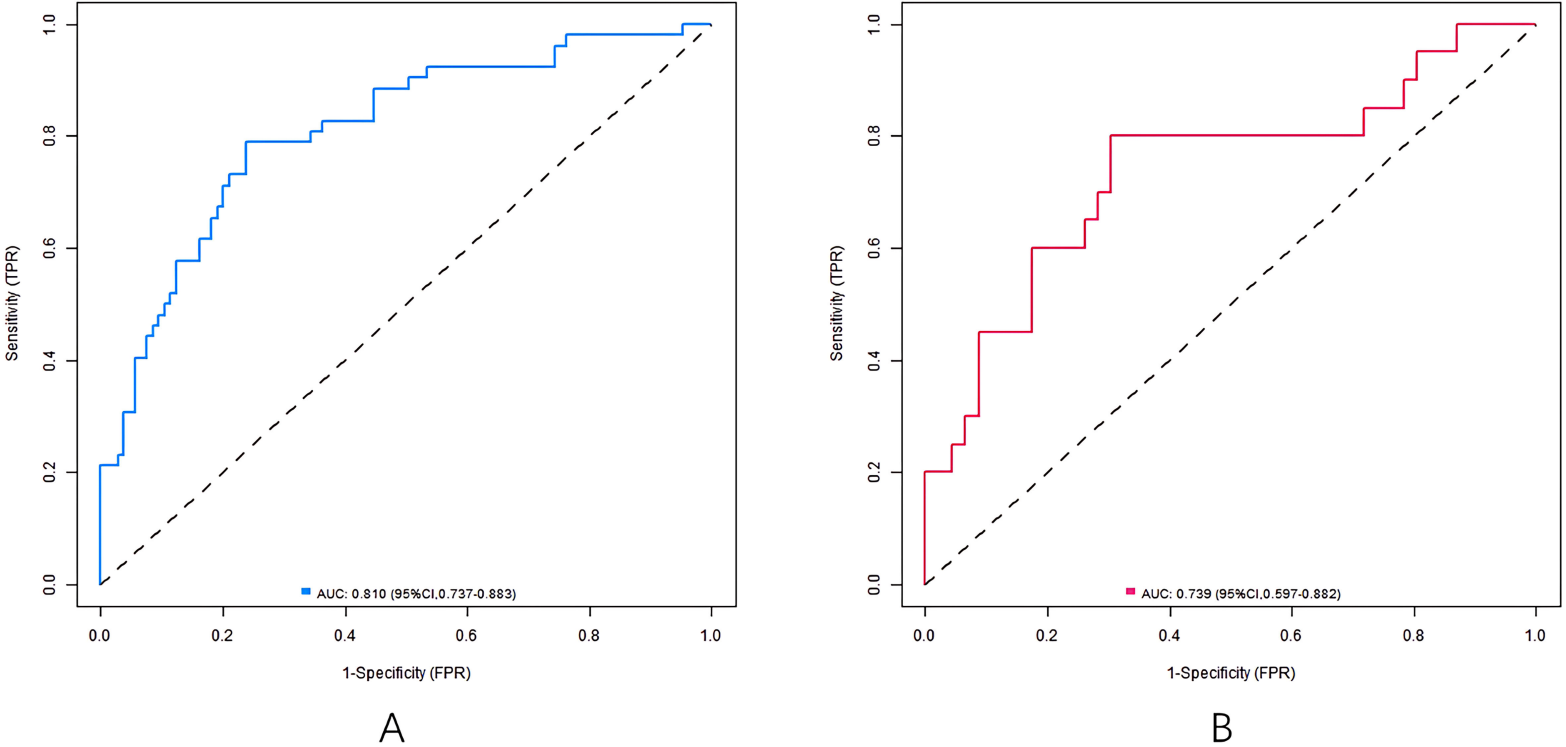
ROC curves in the training set **(A)** and the validation set **(B)**.

The nomogram model has good calibration and fitting degree between the predicted value and the actual value in the training set and the validation set (C-index index is 0.811 and 0.745, respectively. In the calibration curve (**Figure 2**), the mean absolute error between predicted and actual probabilities is 0.160 (training set) and 0.186 (validation set), with *P*>0.05 of Hosmer-Lemeshow test, suggesting good consistency between predicted and actual values. ROC curve shows that the area under the curve (AUC) of training set and validation set nomogram model for predicting hearing impairment in diabetic patients is 0.810 (95% CI: 0.737-0.883) and 0.739 (95% CI: 0.597-0.882), which indicates that the model has good discrimination, and the sensitivity and specificity are 0.788, 0.762 and 0.882, respectively (**Figure 3**).

### 3.5 Decision curve analysis of nomogram prediction model

**Figure 4:**
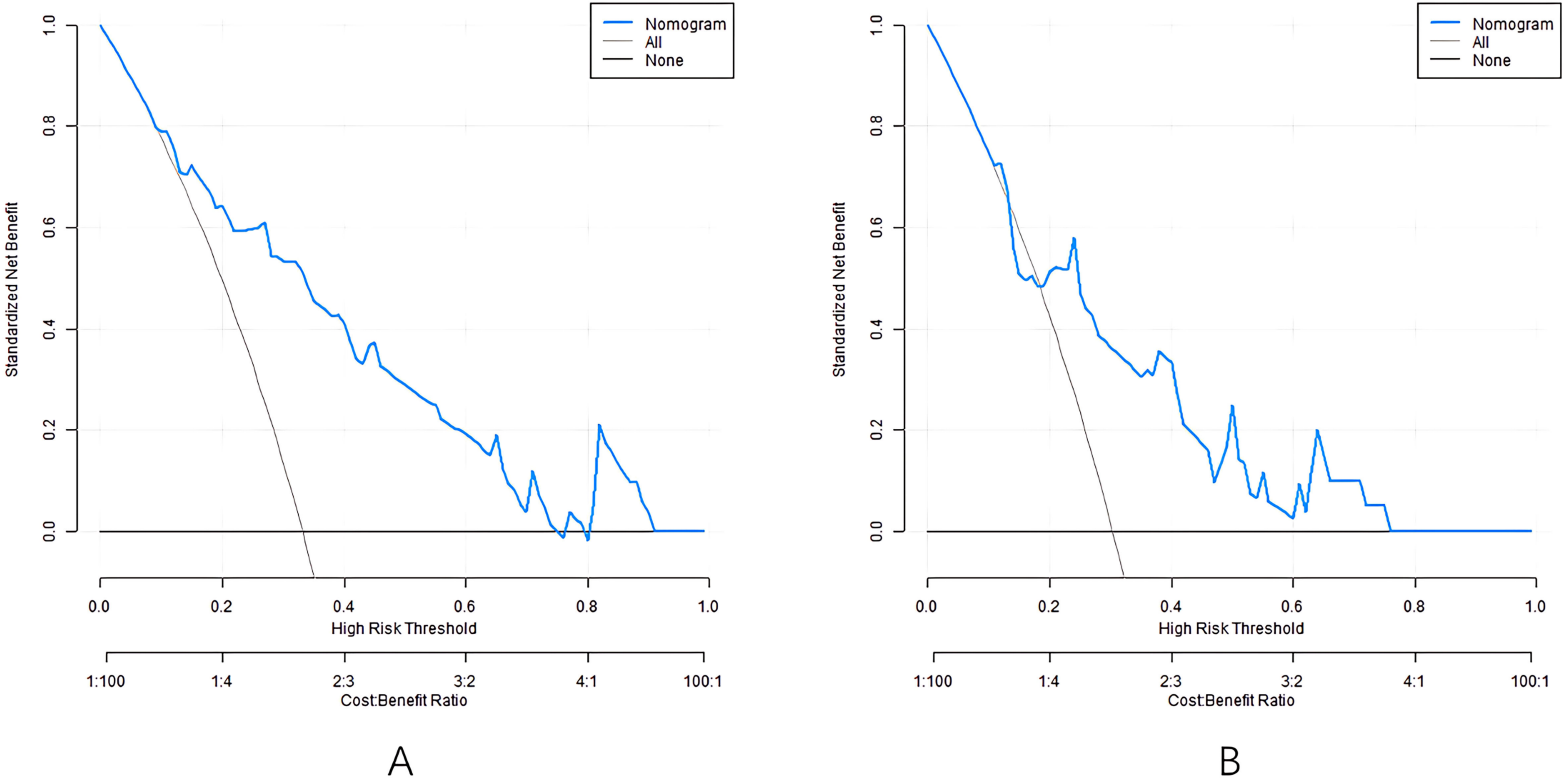
Decision curves in the training set **(A)** and the validation set **(B)**.

The decision-making curve shows that when the threshold probability is about 0.1-0.9, using the nomogram to predict DHI provides more clinical benefits than decisions assuming “all patients have hearing impairment” or “all patients do not have hearing impairment” (**Figure 4**). For example, when the threshold probability is 0.3, the model can avoid missed diagnosis in about 25 out of 100 patients and reduce over-intervention in 15 cases, indicating its practical value in clinically screening high-risk patients.

## 4 Discussion

### 4.1 Development and validation of prediction model

In the study, independent influencing factors of diabetes-related hearing impairment were successfully identified by multivariate logistic regression analysis, and on this basis, a prediction model in the form of nomogram, which included seven predictive factors: diabetic peripheral neuropathy, course of diabetes mellitus, FBG, 2hPG, lncRNA MALAT1, miR-199b and AGEs, was further constructed. Among them, blood biomarkers (MALAT1, miR-199b and AGEs) are the key to improve the discrimination ability of the model: the AUC of the simplified model only including clinical indicators (such as neuropathy, blood sugar and course of disease) is 0.682, while the AUC of the model increases to 0.810 after adding biomarkers (P < 0.05), indicating that biomarkers can significantly improve the prediction efficiency. The simplified model still has a certain reference value (AUC 0.682) for institutions that cannot detect these markers, but its discrimination ability is low.

The prediction efficiency of this model is better than that of similar studies: for example, Al-Rubeaan et al. (7) built a prediction model of hearing loss based on the clinical indicators of patients with type 2 diabetes, and its AUC was 0.68. The retrospective study of Miwa et al. did not build a model, but pointed out the relationship between blood sugar control and hearing protection, and this study further quantified the risk weight of each factor (12). In addition, compared with Yee et al. (13) who paid attention to drug-related hearing loss, this model focused more on the unique biomarkers of diabetes, and was more targeted.

Based on the above influencing factors, this study constructed a nomogram prediction model for DHI. Calibration curve, as another important tool to evaluate the consistency between the predicted value and the actual observation value of the prediction model, in this study, the calibration curve of the nomogram model closely fits the actual value in both the training set and the validation set, and the consistency index (C-index) is 0.811 and 0.745, respectively, and the average absolute error between the predicted value and the real value is 0.160 and 0.186, respectively. At the same time, the results of the Hosmer-Lemeshow test (P = 0 Through decision curve analysis (DCA), the clinical practicability of the model is further evaluated, which provides strong support for the wide application of the model. The results of this study show that the alignment. The net income of the model under different threshold probabilities is higher than that of the model without prediction and only based on risk factors, suggesting that the model can provide important reference for clinical decision-making (14). By using this model, clinicians can more accurately assess the risk of hearing impairment of diabetic patients and make appropriate clinical decisions, so as to minimize the risk of hearing impairment of diabetes and ensure the quality of life of patients (15).

### 4.2 Mechanisms of biomarkers

DHI is a complex pathological process, involving complex pathophysiological mechanisms, including metabolic dysfunction, oxidative stress response, activation of inflammatory mediators and abnormal microvascular structure and function. The results of this study showed that diabetic peripheral neuropathy, AGEs level, FBG and 2hPG level, high expression of LncRNA MALAT1, low expression of miR-199b and long course of diabetes were independent risk factors for DHI. AGEs accumulate in diabetic patients and reach the cochlea via circulation, preferentially depositing in the stria vascularis and spiral ligament (15, 16). Binding to RAGE activates downstream oxidative stress pathways (e.g., NADPH oxidase), causing cochlear vascular endothelial damage and capillary basement membrane thickening, which reduces cochlear blood flow. Impaired stria vascularis function disrupts endolymph ion balance, leading to hair cell degeneration due to insufficient nutrition, ultimately resulting in sensorineural hearing loss predominantly affecting high frequencies (5). lncRNA MALAT1, highly expressed in peripheral blood of patients with DHI, can enter the inner ear through the blood-cochlear barrier and specifically target spiral ganglion cells (9). MALAT1 sponges inhibitory miRNAs (e.g., miR-205-5p), derepressing pro-apoptotic genes (e.g., Bax) and increasing ganglion cell apoptosis. Reduced spiral ganglion cells directly impair auditory signal transmission from hair cells to the central nervous system, causing pathological manifestations such as decreased speech recognition (17). As an important miRNA, miR-199b plays a protective role in many diseases. The results of this study show that the expression level of miR-199b in patients with DHI is significantly reduced, indicating that mir-199b may protect the hearing system from damage by inhibiting the expression of related target genes (18). miR-199b can directly target and bind to the 3’UTR region of IL-1β (seed sequence match: 5’-ACAGUAG-3’), inhibiting its mRNA translation (19). Studies have confirmed that overexpression of miR-199b in the cochlea of diabetic mice can reduce IL-1β levels by 40% and alleviate hair cell damage (20), directly demonstrating its protective effect on the hearing system. miR-199b, a protective microRNA highly expressed in cochlear supporting cells, normally binds directly to the 3’UTR of IL-1β and TNF-α mRNA to inhibit pro-inflammatory cytokine synthesis (7). In diabetes, hyperglycemia-induced DNA methylation silences the miR-199b promoter, and its downregulation leads to excessive release of inflammatory factors, triggering chronic inflammation in the organ of Corti (including inner and outer hair cells). Long-term inflammation damages hair cell stereocilia, causing progressive hearing loss from low to high frequencies (20). The results of this study further confirmed the important role of diabetic peripheral neuropathy in DHI. The levels of FBG and 2hPG are the key parameters to measure the blood sugar control status of diabetic patients. Persistent hyperglycemia accelerates the occurrence and progress of various complications of diabetes by triggering a variety of complex mechanisms including oxidative stress, activation of inflammatory processes and damage to microvascular system (21). The results of this study show that patients with high FBG and 2hPG levels have a significantly increased risk of diabetic hearing loss, suggesting that maintaining good blood sugar management is of vital importance for preventing diabetes-related hearing loss. The duration of diabetes, as a key risk factor for the occurrence and deterioration of diabetic complications, will gradually aggravate the damage of target organs with the continuous extension of the disease course and the persistence of metabolic system imbalance, oxidative stress response and inflammatory process caused by hyperglycemia (22). The results of this study show that the risk of DHI is significantly increased in patients with long-term diabetes, suggesting that long-term diabetes patients should pay more attention to their hearing status (23).

### 4.3 Limitations and future directions

However, this study has several limitations. First, this model is constructed based on a single-center Chinese population, and its applicability may be affected by factors such as ethnicity and regional dietary habits (12). Future multicenter studies with larger, diverse populations (including different ethnicities and diabetes subtypes) are needed to validate universality of the model. Second, we focused solely on LncRNA MALAT1, miR-199b and AGEs as predictors, excluding other potential biomarkers (e.g., 8-OHdG, SLC26A4) and environmental factors (24). The model also wasn’t validated across age, gender or disease duration subgroups. Future research should incorporate additional risk factors and employ advanced methods (e.g., machine learning algorithms) to optimize predictive accuracy. Third, while we evaluated the model’s calibration and discrimination performance, long-term follow-up data are lacking to assess its clinical utility in real-world settings (25). Prospective studies should examine the model’s practical benefits and validate the MALAT1/miR-199b/AGEs interaction mechanisms through *in vitro* experiments. These improvements would enhance the model’s robustness and provide stronger evidence for clinical implementation. In addition, we acknowledge that unmeasured confounders—including prior otitis media history, detailed noise exposure (occupational/leisure), and dietary patterns—could influence hearing outcomes. These factors were not systematically collected in our retrospective design but should be prioritized in future prospective studies.

## 5 Conclusion

To sum up, this study developed and validated a clinically useful nomogram for DHI using peripheral blood levels of LncRNA MALAT1, miR-199b and AGEs. The model enables accurate risk assessment to guide clinical decisions and improve patient outcomes. Future studies should expand sample sizes, incorporate additional risk factors, and conduct long-term follow-up to enhance the model’s precision and reliability.

## Data Availability

The original contributions presented in the study are included in the article/**Supplementary Material**. Further inquiries can be directed to the corresponding author.
