# Supplementary material for: Development and validation of nomogram prediction model for diabetic hearing impairment based on the levels of lncRNA MALAT1, miR-199b and AGEs in peripheral blood
Source: Front Endocrinol (Lausanne). 2025 Sep 10;16:1573661. doi: 10.3389/fendo.2025.1573661 (PMC12457125; doi:10.3389/fendo.2025.1573661)
Supplement: Supplementary file 2 [file Table2.doc]

library(survival)

library(lattice)

library(Hmisc)

library(rms)

log<-read.csv("Data.csv")

dd<-datadist(log)

options(datadist='dd')

summary(fit)

nom<-nomogram(fit）

fun=plogis

fun.at=c(0.001,seq(0.1,0.9,by=0.5),0.999)

plot(nom)

call<-calibrate(fit,cmethod='hare',method='boot',B=1000,data=log)

plot(call,xlim=c(0,1))

fit

library(pROC)

roccurve<-roc(log$Group1~log$External.verification)

plot.roc(roccurve,xlim=c(1,0),ylim=c(0,1))

auc(roccurve)
